# Husbands' involvement in delivery care utilization in rural Bangladesh: A qualitative study

**DOI:** 10.1186/1471-2393-12-28

**Published:** 2012-04-11

**Authors:** William T Story, Sarah A Burgard, Jody R Lori, Fahmida Taleb, Nabeel Ashraf Ali, DM Emdadul Hoque

**Affiliations:** 1 Department of Health Management and Policy, University of Michigan, 48109-2029 Ann Arbor, MI, USA; 2 Department of Sociology, University of Michigan, 48109-1382 Ann Arbor, MI, USA; 3 School of Nursing, University of Michigan, 48109-5482 Ann Arbor, MI, USA; 4 International Center for Diarrheal Disease Research in Bangladesh, GPO Box 128, Dhaka 1000, Bangladesh

## Abstract

**Background:**

A primary cause of high maternal mortality in Bangladesh is lack of access to professional delivery care. Examining the role of the family, particularly the husband, during pregnancy and childbirth is important to understanding women's access to and utilization of professional maternal health services that can prevent maternal mortality. This qualitative study examines husbands' involvement during childbirth and professional delivery care utilization in a rural sub-district of Netrokona district, Bangladesh.

**Methods:**

Using purposive sampling, ten households utilizing a skilled attendant during the birth of the youngest child were selected and matched with ten households utilizing an untrained traditional birth attendant, or *dhatri*. Households were selected based on a set of inclusion criteria, such as approximate household income, ethnicity, and distance to the nearest hospital. Twenty semi-structured interviews were conducted in Bangla with husbands in these households in June 2010. Interviews were transcribed, translated into English, and analyzed using NVivo 9.0.

**Results:**

By purposefully selecting households that differed on the type of provider utilized during delivery, common themes--high costs, poor transportation, and long distances to health facilities--were eliminated as sufficient barriers to the utilization of professional delivery care. Divergent themes, namely husbands' social support and perceived social norms, were identified as underlying factors associated with delivery care utilization. We found that husbands whose wives utilized professional delivery care provided emotional, instrumental and informational support to their wives during delivery and believed that medical intervention was necessary. By contrast, husbands whose wives utilized an untrained *dhatri *at home were uninvolved during delivery and believed childbirth should take place at home according to local traditions.

**Conclusions:**

This study provides novel evidence about male involvement during childbirth in rural Bangladesh. These findings have important implications for program planners, who should pursue culturally sensitive ways to involve husbands in maternal health interventions and assess the effectiveness of education strategies targeted at husbands.

## Background

In Bangladesh, rates of maternal mortality--the death of women during pregnancy, childbirth, or in the 42 days after delivery--are on the decline, but remain very high (194 maternal deaths per 100,000 live births) [1]. The majority of maternal deaths and disabilities occur suddenly and unpredictably between the third trimester and the first week after the end of pregnancy due to hemorrhage, sepsis, and obstructed or prolonged labor [2]. The fact that most deaths occur during childbirth or are the result of an event during childbirth emphasizes the need for every woman to have access to skilled health professionals--such as a doctor, a nurse or a midwife--and adequate health facilities [2]. However, only 27% of all births in Bangladesh are assisted by skilled professionals and only 23% of births take place in a health facility [1].

Women and their families face socioeconomic and cultural barriers to seeking professional delivery care, such as high costs, long distances to health facilities, lack of knowledge about danger signs during pregnancy, and a tradition of using untrained local practitioners during delivery [3]. Two important determinants of care-seeking behavior particularly important in rural Bangladesh are social norms and family dynamics. Cultural norms and beliefs have been shown to delay and sometimes stop women from seeking professional care during childbirth [4]. Some women in Bangladesh do not believe professional medical workers are the best option to manage delivery-related complications [5]. This may be related to the concepts of purity and shame, which keep them away from hospitals and male doctors. Notions of purity are often juxtaposed with pollution in South Asian societies. In the north Indian context, the afterbirth is perceived to be the most severe pollution of all, even worse than defecation or death. There is a similar understanding of childbirth in Bangladesh, such that the person attending the birth must undergo purification after delivering the baby [6]. The mother and newborn are perceived to be in a state of ritual impurity immediately after the birth. In order to prevent other household members from becoming polluted, the birthing woman is confined to a separate room designated for childbirth for seven to nine days following the delivery [7]. Because of the shame and impurity associated with childbirth, women prefer not be in a public space during delivery. Another possible barrier to professional delivery care is the institution of *parda *(or *purdah*) in Muslim culture, which makes a strict separation between men and women and often limits female mobility. Since most doctors in Bangladesh are male, women do not want them to be present during delivery [6].

Family dynamics present another barrier to delivery care in rural Bangladesh. Many individuals in a woman's social network play a role in decisions about reproductive health care utilization, especially when acute problems arise [4,5,8]. Parkhurst and colleagues [5] describe decisions about professional delivery care being made at a crisis point, when a woman's home labor is perceived to be progressing poorly. Close family members in the household, including mothers-in-law and other female in-laws, often give opinions on how to proceed. The same pattern of behavior during pregnancy and childbirth is also found in Nepal, where older women have decision-making authority, especially the mother-in-law [9]. However, the husband is also a possible family member who may influence the decision to seek professional delivery care. It has been suggested that better spousal communication may improve women's maternal health care-seeking behaviors [9,10]. Therefore, husbands' attitudes and beliefs can play a key role in overcoming access barriers to maternal health care in Bangladesh, or conversely, act as barriers themselves [11].

### The husband's role during pregnancy and childbirth

The influence of men in decision-making has been seen in studies of family planning, sexually transmitted infections and HIV, abortion, and infertility. However, there is a relative scarcity of information on men's intentions and practices as they relate to pregnancy and childbirth, especially in South Asia [12]. Some have suggested that male partners act as obstacles when it comes to safe delivery care [13]. However, male involvement during pregnancy and childbirth can lead to positive birth outcomes for the mother and child as well as a healthier marital relationship. A husband's positive involvement can take many forms, including transporting his wife to a qualified provider, providing household money to make that visit, giving helpful informational support during pregnancy, and offering emotional support during labor and childbirth [14].

In South Asia, it has been suggested that women who receive their husbands' approval [15] and women whose husbands are concerned about pregnancy complications [8,16] are more likely to use reproductive health services. However, men are not always encouraged to be involved during pregnancy and childbirth in the South Asian context. For example, men in Nepal are typically discouraged from involvement with pregnancy and childbirth [17,18]. Mullany [18] found that some husbands were interested in supporting pregnancy health, but their lack of knowledge about maternal health posed a significant obstacle to becoming positively involved. Other barriers to the husband's involvement included embarrassment in learning about pregnancy health, work obligations, hospital's restrictions on the husband's entrance into most areas of the hospital, and communication barriers between husbands and wives [18]. It has also been reported that there is a significantly higher probability of male involvement in pregnancy health when men and women made household decisions together [19]. In India, men are traditionally the gatekeepers to health care; however, they know little about pregnancy and childbirth [20]. Chattopadhyay [20] found that men's knowledge about reproductive health and their presence during antenatal care visits were associated with the use of professional delivery care. The studies from Nepal and India suggest that male involvement during pregnancy and childbirth is an important aspect to maternal health care utilization.

In Bangladesh, men often make decisions about women's health care because women are structurally and culturally dependent on men due to their limited mobility and limited educational and economic opportunities [6,11]. This means that even though men may be discouraged from being involved in matters of pregnancy and childbirth, as is the case in the region, their beliefs and perceptions might influence where and how their wives give birth. This study was designed to provide novel evidence about male involvement during childbirth in rural Bangladesh.

### Study aims

This study contributes to the literature on childbirth by examining the husband's role during childbirth from the male perspective. Specifically, this paper aims to (1) better understand the common barriers to delivery care, (2) explore the underlying social factors associated with delivery care utilization, and (3) examine how husbands' involvement during pregnancy and childbirth is associated with the use of professional delivery services.

## Methods

### Setting

A rural sub-district--Durgapur--in the Netrokona district of Bangladesh was selected for this study based on the population's vulnerability to poor health outcomes. Durgapur is a flood-prone area with a population of 198,326 [21]. Durgapur has high rates of poverty, low levels of literacy, poor infrastructure, and limited access to health facilities (approximately one first-level facility per 21,000 people). Durgapur is close to the border with India and has a large number of ethnic families--including Garo, Koch and Hajong--who are often overlooked when healthcare resources are distributed.

### Sample selection

The sampling frame included 10 villages in Durgapur that were selected based on three criteria: (1) the village provided some ethnic diversity to the sample, (2) the village was outside of a five kilometer radius of the nearest health facility, and (3) the village was relatively easy to access given the weather and road conditions. Each village had an approximate population of 1,000. From these 10 villages, 24 households were selected for inclusion in the study using non-proportional quota sampling. First, a total of 12 households that utilized a skilled birth attendant during the birth of the youngest child were selected. This sample was further divided based on the type of skilled birth attendant utilized during childbirth. Skilled birth attendants are typically defined as an accredited health professional, such as a doctor, nurse, or midwife [22]; however, it is extremely difficult to travel to the hospital to find a skilled birth attendant in most rural areas in Bangladesh. Therefore, another type of skilled birth attendant, known as a community-based skilled birth attendant (CSBA), is often used for home deliveries. A CSBA is community-based female fieldworker who has received six months of training in midwifery skills and the identification of maternal danger signs for referral [23].

Our sample included six households that used a doctor, nurse or midwife at a hospital and six households that used a CSBA at home. Each of these 12 households was matched with a household that utilized an untrained *dhatri *(a common term for a traditional birth attendant) during the birth of the youngest child. A *dhatri *is usually an older, non-literate woman who has learned her skills through more experienced *dhatris *[24]. The *dhatris *used by study subjects had received no formal training on childbirth practices. Households were selected based on a set of inclusion criteria, including age of the youngest child (less than one year), age of the mother (18 to 49 years), approximate household income (less than 3,200 Taka [39 USD] per month), and distance to the nearest health facility with at least one doctor, nurse, or midwife on staff (between five to ten kilometers).

The sample was selected by first identifying six women who delivered at one of three local health facilities in Durgapur--two private and one public--using a hospital registry. Next, six women who delivered at home with the help of a CSBA were identified by contacting the CSBAs who worked in the 10 villages in our sampling frame. The interview team visited the household of each woman to determine whether or not she and her husband met the inclusion criteria for the study before starting the interview. Once households utilizing a skilled birth attendant were identified, the interview team went house-to-house to select a household from the same village that met the inclusion criteria, but had used a *dhatri*. Due to misidentification of two birth attendants, two pairs of households were dropped from the final analysis. The final sample is depicted in Table 1.

**Table 1:** Matched Sample (n = 20)

	Skilled Birth Attendant (n)	Untrained *Dhatri *(n)
**Location of delivery **	Hospital (6)	Home (6)
	
	Home (4)	Home (4)

### Data collection

Semi-structured interviews were conducted with both husbands and wives over two weeks in June 2010. Four interviewers (two women and two men) with a background in anthropology and experience in qualitative interviewing were recruited for data collection. All interviewers received two days of training on all of the documents for the interviews (oral script, pre-screening questions, consent form, code sheet, and the questionnaires). All research materials were translated into Bangla. Upon arrival in the field, a pilot test was conducted with two couples (two husbands and two wives) and necessary changes were made.

This paper focuses on the data collected from the interviews with the husbands. Each husband-interview consisted of a ten-question household demographic survey and a 14-question semi-structured interview. All interviews were conducted in Bangla and took place in the couple's home away from family members and other distractions. Interviewers were gender matched with respondents. The husband was interviewed at the same time as his wife, but they were interviewed separately and could not hear or see one another. Interviews with husbands lasted between 40 and 90 minutes (53 minutes on average). The interviewer received written consent to interview and tape record each respondent. The Principle Investigator (PI) was present during half of the interviews with the husbands and supplemented the interviews with field notes describing methodological, theoretical and personal observations during the data collection period. This study was approved by the University of Michigan Internal Review Board (HUM00038924) and the International Center for Diarrheal Disease Research, Bangladesh (ICDDR, B) Ethics Review Board.

### Data analysis

Respondents were de-identified using a numerical code. Interviews were transcribed, translated into English, and analyzed using NVivo 9.0. A combination of open and axial coding was used to identify the properties and dimensions of each major category, or theme [25]. When possible, *in vivo *codes--words which were used by respondents--were used for coding purposes. *In vivo *codes are an important part of sociological research because they pinpoint the meaning of certain ideas or experiences from the respondent's perspective [26]. Intercoder reliability was used to evaluate the validity of the coding categories: two investigators (one Bangladeshi and one American) used the code categories developed by the PI to independently code sections from eight interviews. The average percent agreement among the three coders was 79%.

## Results

The demographic, socioeconomic, and geographic characteristics of the ten husbands whose youngest child was delivered by a skilled birth attendant (doctor, nurse, midwife, or CSBA) were compared to the characteristics of the ten husbands whose youngest child was delivered by an untrained *dhatri *(Table 2). Due to the small sample size, tests for statistical significance across the groups are not very informative, so they are not presented. We display the raw count data and make general observations about differences across the two groups. A larger proportion of households using a skilled birth attendant had only one child and these households appeared to have a higher monthly income per family member compared to households that used an untrained *dhatri*. The higher monthly income per family member may simply be a reflection of the smaller family size among households using a skilled birth attendant.

**Table 2:** Sociodemographic characteristics of men interviewed (n = 20)

Characteristic	Skilled Birth Attendant (n)	Untrained *Dhatri *(n)
Age of respondent (years)		

20-29	5	4

30-39	5	3

40 +	0	3

Age of youngest child (months)		

< 1	1	0

1-5	8	6

6 +	1	4

Number of children		

1	5	0

> 1	5	10

Child deaths		

None	7	6

1-2	3	4

Education		

None	2	5

Class 1-4	5	2

Class 5-9	3	3

Occupation		

Agricultural laborer	7	6

Other	3	4

Ethnicity		

Bengali	8	8

Garo	1	1

Hajong	1	1

Religion		

Muslim	7	7

Hindu	2	2

Christian	1	1

Approximate monthly income per household (Taka and USD)		

1,000-2,499 [12-31 USD]	4	4

2,500-3,200 [32-39 USD]	6	6

Approximate monthly income per family member (Taka and USD)		

250-649 [3-8 USD]	4	8

650-1,100 [9-13 USD]	6	2

Distance to closest health facility (km)		

5-6.9	7	6

7-10	3	4

Each husband's interview was coded in two ways: (1) themes that were similar across all respondents, or *common themes*, and (2) themes that differed according to the type of provider utilized during childbirth, or *divergent themes*. We first present three common themes, or barriers, related to delivery care before presenting the underlying, divergent factors related to delivery care, including husbands' provision of social support and perception of social norms related to childbirth.

### Common themes

The interviews with husbands revealed a number of common themes related to the decision about whether or not to seek professional delivery care. Each theme was found in each of the interviews, irrespective of the type of provider.

### Poor transportation

Each household in the sample was at least five kilometers away from the nearest health facility equipped to perform deliveries. The condition of roads and availability and cost of local transportation were mentioned as barriers to hospital-based deliveries. There are only 18 kilometers of *pucca *roads (good quality, black-topped roads) in all of Durgapur. The remaining roads are dirt, which turn into mud during the rainy season. Nine husbands mentioned the condition of the roads as a major barrier to using health facilities.

*Everything depends on transportation. People don't go [to the hospital] very often since they need to hire three to four boats [to cross the rivers]. Also, the condition of the road is very poor. If the pregnant woman travels by rickshaw or motorcycle or push car, then she may get hurt and face more problems*. - 26-year old husband, CSBA home delivery

Local transportation is often perceived as too expensive and hard to find. Types of transportation available in Durgapur include (from most to least expensive) the *tempo *(a small three-wheeled motorized vehicle), motorcycle, boat, rickshaw, push cart, and walking. Many families need to use more than one form of transportation when traveling to the hospital, especially during the rainy season.

### Lack of money

Another major barrier to utilizing professional health services are direct costs--such as the cost of transportation, medicine, and hospital fees--and indirect costs--such as opportunity costs related to leaving household responsibilities and taking time away from work. The costs associated with the utilization of professional delivery care are often equated to the cost of selling one's land or forfeiting one's livelihood.

*It's a money matter. [People in our village] can't go [to the hospital] due to the scarcity of money. So if we can solve it in the village, if Allah wants it like this, we don't need to go. So we delay the matter and observe the [birthing] situation. Then, if the problem is serious, we go to the clinic. But it's almost like solving the problem by selling your land*. - 43-year old husband, *dhatri *home delivery

### The danger of childbirth

The perception that one is not susceptible to complications during childbirth could act as a barrier to the utilization of professional delivery care, but childbirth was perceived as risky by all of the husbands interviewed. Most agreed that complications could arise during delivery, but this belief did not always lead to the utilization of a skilled birth attendant during delivery.

*Definitely [childbirth] is dangerous. After conceiving a baby it becomes dangerous. There is tension that anything can happen at any time. There is tension about whether the baby will be born healthy or not*. - 25-year old husband, hospital delivery

*To me, it is a risky thing.... Both mother and child can die. Sometimes it is seen that the baby dies and the mother is alive. Again, it is seen that the mother dies and the baby is alive. If it is normally done, then it is done. But, if not then there is a lot of suffering*. - 28-year old husband, *dhatri *home delivery

### Divergent themes: social support and perceived social norms

There were many themes identified through the open coding process that provided insight into the unique role of husbands during childbirth, in addition to the themes above that showed similarity across respondents. However, our goal was to focus on themes that (1) differed between husbands whose wives utilized a skilled birth attendant and those whose wives utilized an untrained *dhatri*, and (2) were grounded in sociological and psychosocial theories related to health care utilization. The two themes that met these two criteria were social support during labor and delivery and perceived social norms related to delivery.

### Social support

Social support is the term used to describe the functional content of social relationships. Social support differs from other functions of social relationships because it is consciously provided by the sender and is intended to be helpful to the receiver. This distinguishes it from other forms of social influence that are intentionally negative or passively experienced by the receiver. Social support often attempts to influence the behaviors of the receiver in a caring, trusting, and respectful context [27]. Social support can be categorized into four broad types of supportive behaviors: emotional support, instrumental support, informational support, and appraisal support. We draw upon the first three categories of social support--emotional, instrumental, and informational--to describe the type of support provided by husbands to their wives during childbirth. However, there were also situations where the husband did not provide any of these types of support to his wife during the labor and delivery period.

### Emotional support/involvement

Emotional support involves the provision of empathy, love, trust and caring [27]. However, in order for emotional support to have a positive influence it must be perceived as helpful by the recipient [28]. The majority of husbands whose wives utilized professional delivery care exhibited some level of emotional involvement with their wives during pregnancy and childbirth. However, a husband's emotional involvement was not always perceived as supportive by his wife. Therefore, we will refer to this theme as emotional involvement. Emotional involvement was often found in the form of prayer, which is a way of expressing love or caring when the husband is not physically present. However, emotional stress, such as worry and tension, was also coded as emotional involvement. A husband's stress during labor and delivery showed a level of emotional investment that often led to other supportive behaviors. The tension and worry usually arose from the husband's perception that his wife was at risk for a complication during delivery or a previous experience with the death of a child or family member during delivery.

*In my mind I felt there were some problems. I was worried. I thought I should go [to see her] earlier. I wanted to observe what happened. I could be present on the spot. I am her husband and she is my wife. That is why I was interested to come [home] early; otherwise I would come the next morning*. - 26-year old husband, CSBA home delivery

*How could I sleep! The light was on and I was awake for the whole night.... At first I was not thinking about the hospital, but later, when I was tense, I took her [to the hospital] the next day*. - 35-year old husband, hospital delivery

### Instrumental support

Instrumental support involves the provision of tangible assistance that directly helps a person in need [27]. Husbands whose wives utilized professional delivery care, and those that utilized an untrained *dhatri*, each provided some level of instrumental support. However, the type of instrumental support differed depending on whether the delivery took place in a hospital or at home. In all cases the husband typically provided instrumental support to his wife once she started the first stage of labor. Husbands whose wives delivered at a hospital often arranged transportation and collected money for the hospital fees. Husbands whose wives delivered at home provided support by calling the birth attendant or going to the birth attendant's home to inform her of the impending delivery. Husbands whose wives delivered at home often knew the birth attendant personally (i.e., she was a family member or an acquaintance from the village).

*Yes, I called. I went there to call *chachi *(father's brother's wife) saying, '*Chachi, *I have a problem. The placenta is not removed.' Then she asked, 'Why? What is the problem? Was there not any *dhatri *in your home? No *dhatri?' *I said that the *dhatri *was there and [she] asked [me] to bring you here. Then she said, 'You go, I am coming.' When I saw that she was late, then I went to her again*. - 31-year old husband, CSBA home delivery

Some husbands were able to arrange resources to pay for the transportation and hospital fees. Although public hospitals are free of charge, the majority of the respondents preferred the private fee-for-service health facilities. There were two exceptional cases where a husband saved money to pay for an ambulance and where a husband used his land as collateral to repay a loan for hospital fees. Typically, the husband collected money from neighbors and relatives during the first stage of labor.

Husbands whose wives needed to travel to the hospital for delivery were also involved in coordinating or providing the transportation.

*She has a younger brother. Though we rented a *thelagari *(push car), her brother and I pulled it to take her to the hospital. We admitted her in the hospital and I was there all the time*. - 31-year old husband, hospital delivery

Additionally, husbands prepared food for their wives and other visitors as well as purchased medicine for their wives.

*I explained the whole situation [to the village doctor] that my wife could not deliver the baby in spite of severe pain. Then he suggested some medicine and I brought the medicine for her*. - 32-year old husband, *dhatri *home delivery

### Informational support

Informational support involves the provision of advice and suggestions that a person can use to address a problem [27]. Informational support was typically provided by husbands whose wives utilized professional delivery care at home or at the hospital. In our context, informational support refers to the active provision of information from the husband to his wife. The husband provided his opinion about the type of provider his wife should use during delivery and in some cases he made the final decision about the type of provider. Some husbands in our study planned in advance to go to the hospital or to call a CSBA when the labor pains started. However, most husbands asserted their opinion when they perceived the labor to be progressing poorly.

*I said to my wife, 'If you have doubt in your mind and if you face trouble, then you can be admitted to the hospital...' She said, 'You pray for me and keep faith in Allah.' My intention was to take her to the hospital if she faced any trouble*. - 26-year old husband, CSBA home delivery

*... after [my wife was in labor] the whole night, in the morning at 8 am, I said [the delivery] will not work at home. We have to go to the doctor. Then, in a hurry, I called the [push car] and then took her to the hospital. By the blessing of Allah I found the doctor when we reached [the hospital]. - *35-year old husband, hospital delivery

### Uninvolved during childbirth

Not all of the husbands were involved in providing social support during the delivery of their youngest child. Husbands whose wives utilized an untrained *dhatri *at home were typically not involved in any aspect of the labor and delivery process. Husbands who were uninvolved did not necessarily make a conscious decision to be absent during childbirth. Many factors could have contributed to a husband's absence, including his wife's lack of knowledge about the labor process or a general lack of communication between spouses. Lack of involvement appeared in three different forms: (1) the husband's passive agreement with his wife's decision with no input of his own; (2) the lack of communication between the husband and wife during pregnancy, such that the husband was unaware of the timing of the delivery; and (3) the husband's belief that his presence was not necessary once labor started and female family members took control.

*When they tell me to bring [the *dhatri], *I have to bring her. I have to consider her a good nurse*. - 43-year old husband, *dhatri *home delivery

I: Were you there when the pain started?

*R: No, I went for work.... Later the news reached me that the baby [was born]*.

I: Why did you go away? Didn't you know that the baby would be born that day?

*R: We don't know about this a lot. The woman knows about this matter. (Laugh)" - *48-year old husband, *dhatri *home delivery

*R: I didn't have to do anything. I called my younger sister. Then my older sister came and after her arrival the delivery was over within half an hour*.

I: Didn't you hear about when the labor started?

*R: I didn't know that it was time to deliver. I went to the marketplace*. - 28-year old husband, *dhatri *home delivery

### Perceived social norms

The second divergent theme on which we focused was the husband's perception of social norms related to childbirth. Social norms can influence the type of support a husband provides his wife during delivery. According to Lewis and colleagues [29], social norms are "... expectations held by social groups that dictate appropriate behavior and are thought of as rules or standards that guide behavior" (p. 254). In Bangladesh, perceived social norms of the husband are important to his wife because of their close social relationship and because of his decision-making power within the household. There are many social norms related to childbirth in Bangladesh; however, this study focuses on one in particular--the medicalization of childbirth.

### Medicalization of Childbirth

The medicalization of childbirth refers to the redefining of physiological reproductive processes as biomedical problems that can be treated by the medical profession [30]. This view differs from the traditional view in Bangladesh, in which childbirth is perceived as a natural process that should be experienced at home according to local customs [6]. In many Western countries the medicalization of childbirth is often associated with unnecessary biomedical interventions. However, the idealization of "natural childbirth" can often lead to major complications for women in developing countries who do not have access to emergency obstetric care. Therefore, in the context of this study, the medicalization of childbirth refers to a contemporary view, which holds that timely access to professional medical care is necessary to prevent the primary causes of maternal mortality in the event of problems during delivery.

The concept of medicalization is particularly relevant in Bangladesh, a country with a history of medical pluralism. In rural Bangladesh, indigenous medical traditions exist parallel to contemporary, allopathic medical systems. Deciding which type of provider to use during a medical emergency is a complex process, which requires the input of many influential individuals [31]. For the purpose of this study, the perception of medicalization of childbirth among the husbands emerges in the contrast between *old day *and *modern age *thinking. These *in vivo *codes--terms taken from or derived directly from the language of the informant--came from an interview with a husband whose wife delivered at home with the assistance of a trained CSBA.

I: What if the delivery is done at home?

*R: I suppose home is preferred by the elderly people of old day. In this consideration, it is better not to keep the expectant [mother] at home*.

I: What are the problems that can be faced at home?

*R: It is not possible to do everything at home. Women who are ignorant and not up-to-date think that the delivery is always done normally. It is foolish if we don't keep pace with the modern age. In present circumstances, it is better to contact the doctor. A nurse is needed to stay beside the patient all the time in order to look after her meals and medicine. - *26-year old husband, CSBA home delivery

Husbands who were coded as "old day" along the medicalization spectrum mentioned expectations of "normal" or "natural" deliveries at home. They also described traditional practices during childbirth and their reluctance to involve a doctor. Many of these husbands described the pressure from other family members, including their wives, for a home delivery. The majority of husbands whose wives utilized an untrained *dhatri *at home displayed old day thinking.

I: Which [place] do you prefer most [for delivery]; at home or at the hospital?

*R: If [my wife] is comfortable at home, then at home. I don't find any logic in communicating with a doctor. If she is well at home, then home is better*. - 43-year old husband, *dhatri *home delivery

*I: Why did you apply *telpora *(sacred oil)?*

*R: *Telpora *was at home. After applying it, pain develops and [the delivery] won't be hard*.

*I: How did you come to know [that you should use *telpora]*?*

*R: *Kabiraj *(traditional practitioner) told me. He was my grandfather and he told me to [use *telpora] *like this, and then the baby will be born*. - 32-year old husband, *dhatri *home delivery

Husbands who were coded as "modern age" mentioned the importance of going to the hospital for delivery, technology at the hospital, and consulting a doctor during pregnancy. One respondent was a migrant laborer who traveled to the capital city for work and brought modern ideologies back to his village. Others had close social connections to trained health providers or to non-governmental organizations, which promoted a biomedical approach to delivery care. The majority of husbands whose wives utilized professional delivery care displayed modern age thinking.

*I: Aren't nuns and *dhatris *doing [deliveries] as well?*

*R: No, they are from old time. Now time has changed. We are not in the old era now. Now the trained people are doing their jobs.... Suppose, if the patient's condition became complicated, [the family would] carry them to the hospital. And doing delivery by the nurse is better*. - 35-year old husband, CSBA home delivery

*Everything can be found [in the hospital]. Nothing can be found in the village. In the hospital, they gave medicine to the baby. Can they do that in the village? *- 35-year old husband, hospital delivery

## Discussion

The majority of the literature on the utilization of professional delivery care in low-income countries focuses on the barriers encountered by women [32]. However, the role of the family, in particular the husband, during pregnancy and childbirth is important to the understanding of women's utilization of professional maternal health services [8,14-20]. This study was designed to look beyond the common barriers to the use of professional delivery care in Bangladesh and examine the underlying factors related to husbands' involvement (or lack of involvement) in delivery care utilization.

In order to examine the underlying factors, we purposefully selected households that differed by the type of provider utilized during delivery. Therefore, the themes and characteristics that were common across all respondents, and did not vary with the type of provider utilized during delivery, were eliminated as sufficient barriers to the utilization of professional delivery care [33]. The findings revealed three such themes common across households: (1) poor transportation, (2) lack of money, and (3) the perceived danger of childbirth. Although these have been shown to be necessary barriers to the utilization of professional delivery care [32], we suggest that they are not sufficient. In addition, we designed the sample so that socioeconomic and geographic characteristics were common among households that utilized professional delivery care and those that did not. Although many of these characteristics (e.g., income, education, and distance to the health facility) have been shown to be correlated with the utilization of professional delivery services [2,3,32,34], they are not sufficient to explain the type of provider utilized during delivery among our respondents.

Our findings suggest that husbands' provision of social support to their wives and perceived social norms vary systematically with the type of provider, which implies that these factors are important to understanding delivery care utilization in Bangladesh. In order to better understand husbands' social support and perceived social norms, these two factors were used as the axes of our analysis (Table 3). The two ends of the axis of social support were *involved *(which included emotional, instrumental, and informational support) and *uninvolved*. Perceived social norms were divided into two categories based on husbands' perceptions of the medicalization of childbirth: *old day *and *modern age *thinking. These two categories were not mutually exclusive; that is, some husbands displayed examples of old day and modern age thinking in the same interview. Based on the analysis, husbands whose wives utilized a skilled birth attendant were more involved and modern age thinkers. That is, these husbands provided emotional, instrumental and informational support to their wives during delivery and believed medical intervention was necessary during childbirth. On the other hand, husbands whose wives utilized an untrained *dhatri *at home were generally uninvolved and old day thinkers. That is, these husbands were uninvolved during delivery and believed childbirth should take place at home according to local traditions. There was no distinguishable pattern for husbands who were uninvolved and modern age thinkers or husbands who were involved and old age thinkers.

**Table 3:** The axes of social support and perceived social norms

		Social Support	
		**Involved**	**Uninvolved**

**Social Norms**	**Modern Age**	Skilled Birth Attendant	Inconclusive
	
	**Old Day**	Inconclusive	*Dhatri*

We identified two potential rival hypotheses that may explain the variation in the type of delivery care utilized: (1) women whose deliveries appear to be normal are more likely to stay home, while women whose deliveries appear to be progressing poorly are more likely to go to a hospital, and (2) women who only have one child are more likely to use professional delivery care, while women who have more than one child are more likely to stay home. In order to determine if the first rival hypothesis was true, we examined the differences between women who delivered at home with a CSBA to women who delivered at home with an untrained *dhatri*. This analysis allowed us to eliminate the potential effect of complications among women who delivered at a hospital. That is, women who delivered at home with a CSBA and women who delivered at home with an untrained *dhatri *encountered similar levels of perceived and actual complications. We found similar coding patterns on the two axes of analysis (social support and perceived social norms) when husbands whose wives utilized professional delivery care at a hospital were excluded from the analysis.

The second potential rival hypothesis originated from the fact that parity differed substantially by the type of provider utilized during childbirth (Table 2). This association may be due to the fact that the first birth can be more difficult for a woman who has no previous delivery experience. It has been shown in South Asia that a high value is often placed on the first pregnancy and the woman's family helps her get the best care possible [35]. Although this is certainly a plausible explanation for some of the differences between the two groups, the same coding patterns existed when husbands of uniparous women were excluded from the analysis.

The results from this study provide a new way to conceptualize a husband's involvement in his wife's decision to seek professional delivery care in rural Bangladesh. Lewis and colleagues [29] explain how influence and communication between interacting partners can affect each other's motives, preferences, behaviors, and health outcomes. Specifically, our study suggests that a husband's social support and social norms are associated with his wife's use of delivery care. Rozario [6] explains how the husband is often responsible for making decisions about health care during childbirth in Bangladesh. Since social norms become more salient when the receiver of social influence is motivated to maintain a relationship with the sender of social influence [36], women are likely to be receptive to their husbands' beliefs and opinions about delivery care. These findings are supported by previous studies that suggest male involvement in pregnancy and childbirth and good spousal communication are associated with increased use of maternal health services [8-10,18-20].

This study was not designed to account for all of the factors involved in the decision-making process, nor was it designed to account for the perspective of everyone in a woman's social network. Moreover, our view of the social processes explored in this study was based solely on the husband's report. Future qualitative research should explore the role of broader social networks, including other key family members and other influential people, in the decision-making process during childbirth. It should also consider comparing husbands' and wives' perspectives when making decisions about the use of professional delivery care. Furthermore, while this study provides a comparative picture of husbands' involvement during childbirth, it was not designed to infer a causal association between husbands' involvement and the use of professional delivery care. Since we used retrospective accounts it is difficult to determine whether husbands' beliefs and opinions about childbirth were formed prior to the delivery of their youngest child or afterward.

### Limitations

While this study provides novel information and rich detail, an important limitation is the potential endogeneity of husbands' involvement in the decision to utilize professional delivery care. There could be a reverse causality problem, such that husbands were more likely to be involved because their wives utilized professional delivery care, rather than the reverse association that forms the focus of our analysis and interpretation. In order to address this potential endogeneity problem, future qualitative studies could use a prospective study design to follow husbands and wives during pregnancy and delivery to better understand the timing of events related to the husband's level of involvement. Additionally, the short fieldwork period did not allow for time to follow-up with the participants to clarify any misunderstandings. In particular, the sample size was reduced due to misunderstandings about who actually delivered the child. Furthermore, the qualitative methods that we used when talking to husbands did not allow us to capture details about personal, psychological, and temperamental differences that may have an impact on husbands' involvement during pregnancy and childbirth. Finally, we could not eliminate potential sources of measurement error, such as interviewer effects and retrieval failure. Possible interviewer effects include the respondent answering a question with information that he believes the interviewer desires to hear, or what he thinks is "correct." Retrieval failure occurs when the events were never coded in the respondent's memory due to the lack of significance of the event or because the respondent was not present during the events in question.

## Conclusions

The findings presented in this paper suggest that the level of social support provided by the husband to his wife and the husband's perception of social norms related medical intervention during childbirth are both associated with the type of provider utilized during childbirth. The husband's perception of social norms related to professional care is important to the type of advice and support he will give his wife during pregnancy and childbirth, which has important implications for the development of future maternal health interventions.

Areas of fruitful future research include the further elucidation of the types of social support provided by the husband and the critical social norms related to childbirth in Bangladesh. This and future research could be used to create valid and reliable measures to assess the quantitative impact of social support and social norms on the utilization of professional maternal health services using a more representative sample. These findings may also be useful to program planners, who should pursue culturally sensitive ways to involve the husband in maternal health interventions and assess the effectiveness of education strategies targeted at husbands.

## Competing interests

The authors declare that they have no competing interests.

## Authors' contributions

WTS designed the study, participated in data collection, and drafted the manuscript. SAB and JRL contributed to the study design and critically revised the manuscript. FT and NAA led the data collection process and made revisions to the manuscript. DMEH facilitated ethical approval, coordinated field visits, and made revisions to the manuscript. All authors read and approved the final manuscrpit.

## Pre-publication history

The pre-publication history for this paper can be accessed here:

http://www.biomedcentral.com/1471-2393/12/28/prepub
